# Supplementary material for: DJ-1 protects proteins from acylation by catalyzing the hydrolysis of highly reactive cyclic 3-phosphoglyceric anhydride
Source: Nat Commun. 2024 Mar 5;15:2004. doi: 10.1038/s41467-024-46391-9 (PMC10915168; doi:10.1038/s41467-024-46391-9)
Supplement: Supplementary file 1 — Supplementary Information [file 41467_2024_46391_MOESM1_ESM.pdf]

Supplementary information for:

DJ-1 protects proteins from acylation by catalyzing the hydrolysis of highly reactive cyclic 3-phosphoglyceric anhydride

Akhmadi et al.,

Supplementary information table of content:

Supplementary Figure 1.
Supplementary Figure 2.
Supplementary Figure 3.
Supplementary Figure 4.
Supplementary Figure 5.
Supplementary Figure 6.
Supplementary Figure 7.
Supplementary Figure 8.
Supplementary Figure 9.

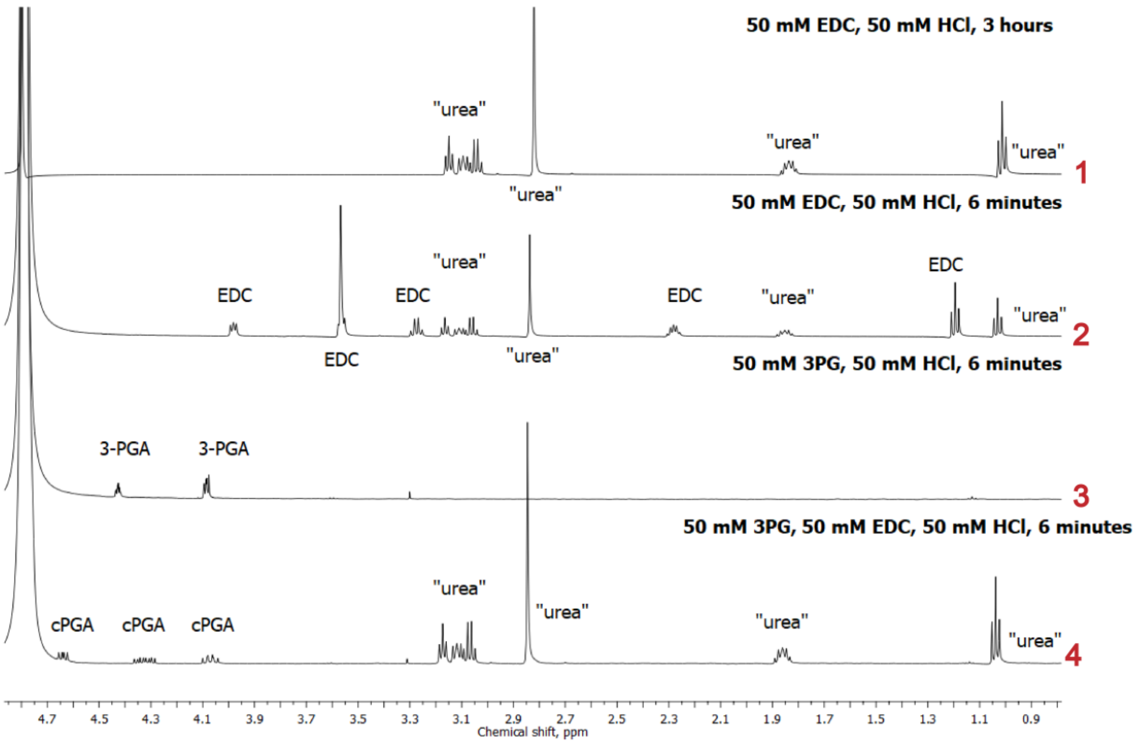

Supplementary Figure 1. EDC-assisted cyclization of 3PG. Comparison of ^{1}H-NMR spectra of EDC after 3-hour (1) and 6-minute (2) incubation in 50 mM HCl/D_2O allows assigning proton peaks to EDC and its urea-like hydration product (labelled as “urea”). Comparison of ^{1}H-NMR spectra of 3PG (3) and 3PG+EDC (4) in 50 mM HCl/D_2O six minutes after the start of reaction reveals that all of the 3PG has been converted to cPGA and all of the EDC has been converted to its urea-like derivative. Note that no other products are present in sample 4, indicating that this reaction occurs with near 100% yield. This experiment was repeated at least 5 times with similar results.

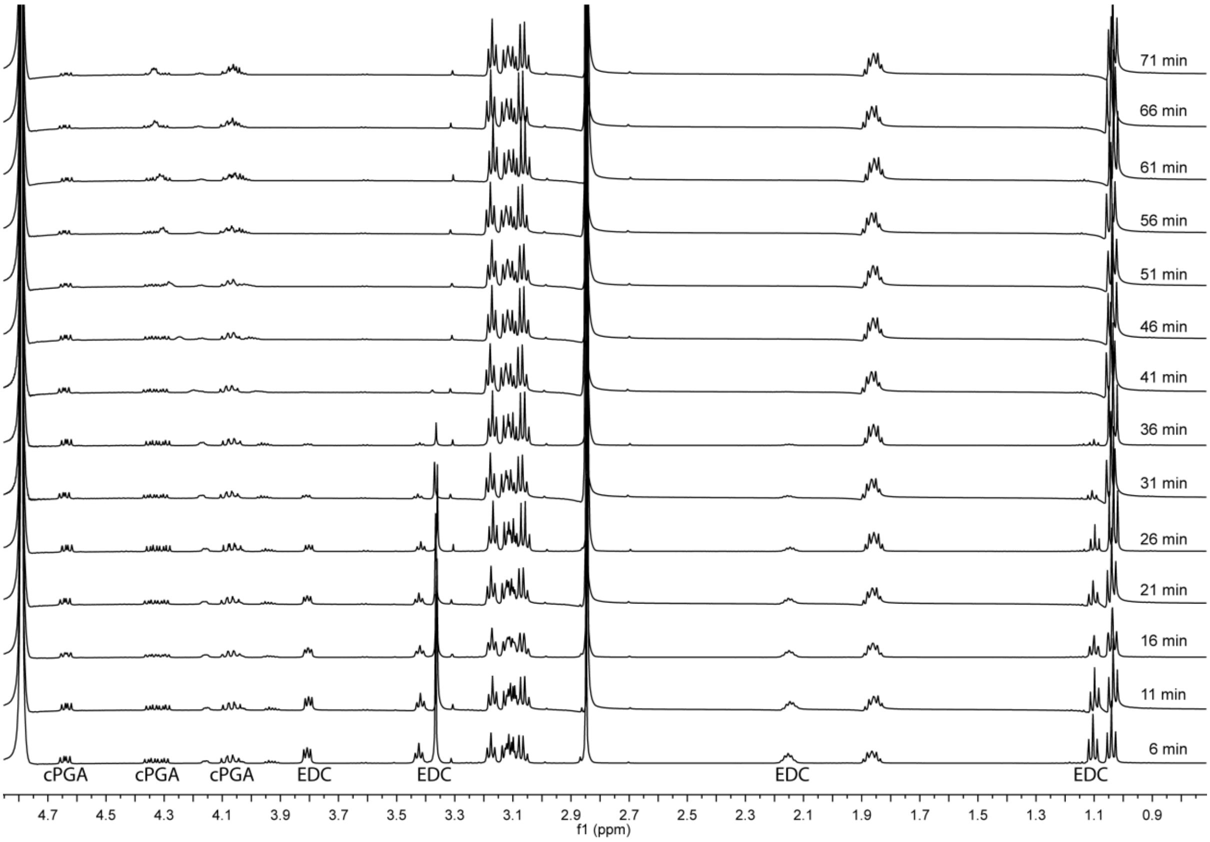

Supplementary Figure 2. Time course of cPGA decomposition in the presence of excess of EDC. ^{1}H-NMR spectra of a mixture of 50 mM 3PG and 60 mM EDC in 50 mM HCl/D_2O were recorded every 5 minutes. Note that cPGA signals start decreasing shortly after the disappearance of EDC at 36-41 minutes. Unlabeled peaks at the beginning of the reaction correspond to the hydration product of EDC. This kinetic series was reproduced twice with similar results.

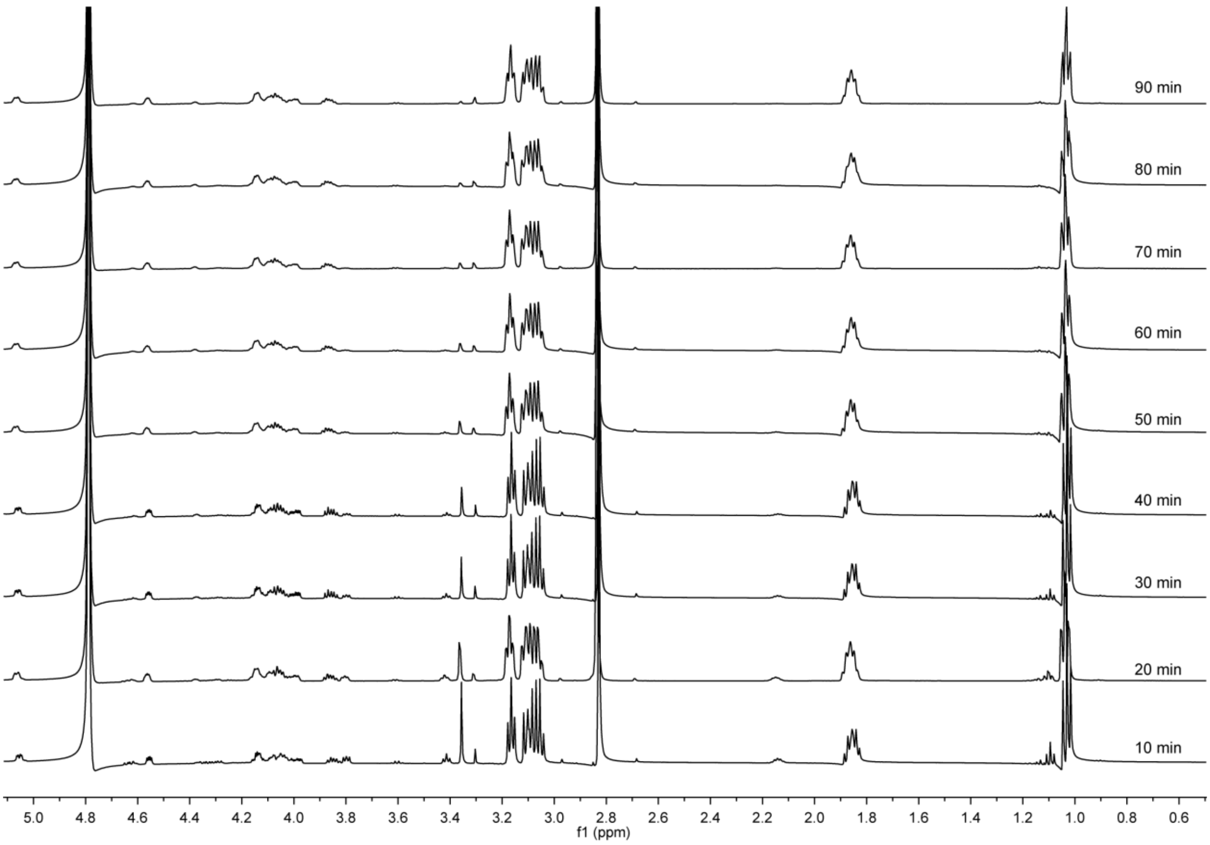

Supplementary Figure 3. Time course of cPGA decomposition at neutral pH. ^{1}H-NMR spectra of a mixture of 50 mM 3PG and 50 mM EDC in 50 mM HCl/D_2O were recorded every 10 minutes after neutralization of reaction mixture. This kinetic series was reproduced twice with similar results.

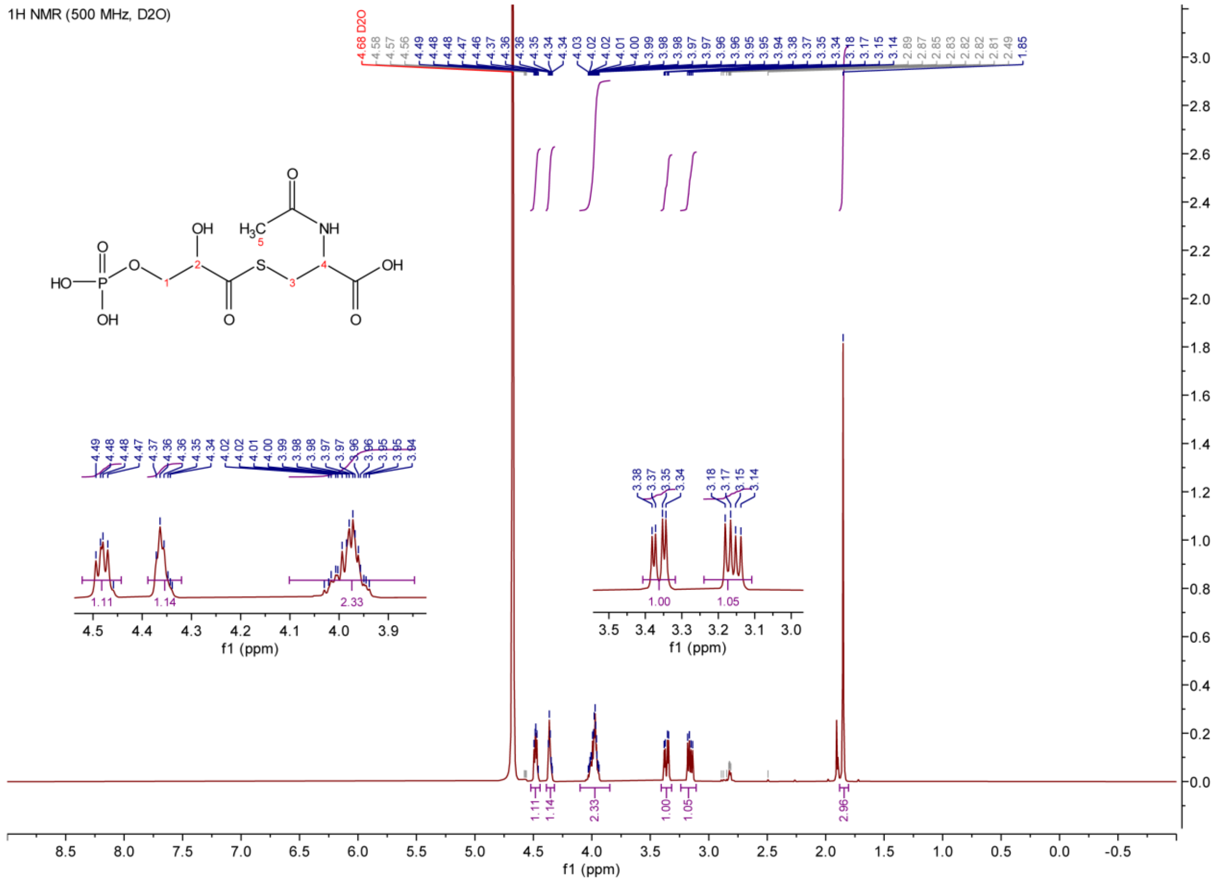

Supplementary Figure 4. ^{1}H-NMR spectrum (500 MHz, D_2O) of isolated N-acetylcysteine thioester of 3-phosphoglyceric acid. δ 4.48 (dd, 1H, J = 7.3, 4.8 Hz, H4), 4.41 – 4.32 (m, 1H, H2), 4.06 – 3.92 (m, 2H, H1′ and H1″), 3.36 (dd, 1H, J = 14.3, 4.8 Hz, H3′), 3.16 (dd, 1H, J = 14.3, 7.3 Hz, H3″), 1.85 (s, 3H, H5). Expanded spectrum regions from 4.5 – 3.9 ppm and from 3.5 – 3.0 ppm are shown to reveal the details arising due to spin-spin coupling.

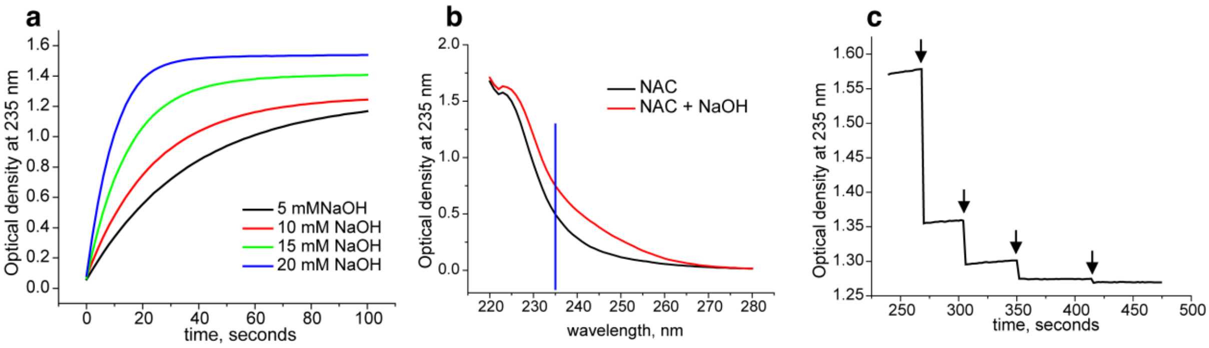

Supplementary Figure 5. Development of spectrophotometric cPGA assay. All experiments were conducted in 50 mM phosphate buffer (pH 7.0). **a** After mixing of 0.5 mM cPGA with 5 mM of NAC, samples were titrated with NaOH to accelerate the reaction through deprotonation of the thiol group of NAC. The addition of 20 mM NaOH immediately after NAC is sufficient for the reaction to reach a plateau in ~20 seconds. The addition of 20 mM of NaOH shifted the pH to ~8.2. A more basic pH would likely accelerate the reaction even further but would also increase the likelihood of spontaneous hydrolysis of cPGA and thioester. **b** UV absorbance spectrum of 5 mM NAC in the presence and absence of 20 mM NaOH. Deprotonation of NAC by NaOH leads to an increase in the absorbance likely due to stronger absorption by the thiolate anion of NAC. Horizontal blue line is drawn at 235 nm. **c** After reaching plateau levels following derivatization with NAC, sample was titrated back with HCl (arrows indicate addition of 20 mM HCl) to the point where no decrease in absorbance due to protonation of NAC was observed. All figures are representative of at least three independent experiments.

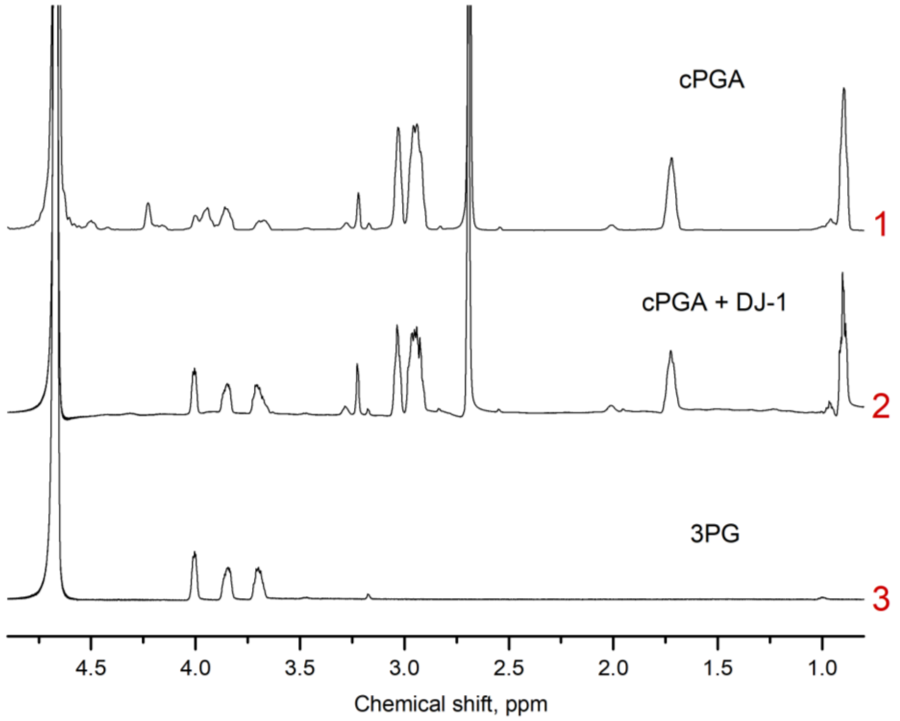

Supplementary Figure 6. DJ-1 converts cPGA into 3PG. 50 mM cPGA in D_2O was diluted with 50 mM phosphate buffer in D_2O to a final concentration of 6 mM in the absence (1) or presence (2) of 50 µM of DJ-1 followed by recording of ^{1}H-NMR spectra. Comparison to the ^{1}H-NMR spectrum of 3PG recorded under the same conditions (3) reveals that DJ-1 completely converts cPGA into 3PG, while cPGA decay results in additional decomposition products. This experiments is representative of at least three independent experiments

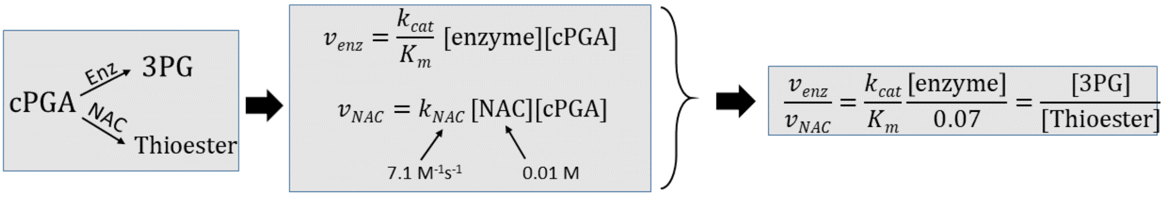

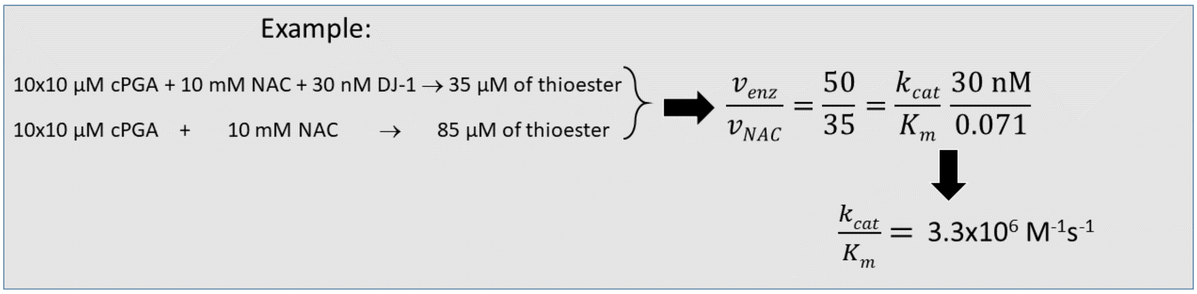

Supplementary Figure 7. Competition assay to determine k_{cat}/K_m. For any two parallel (competing) reactions of the same order, the ratio of concentrations of products is the same as the ratio of respective reaction rates. Keeping [cPGA] in the low µM range by multiple sequential additions (e.g. 10x10 µM) is necessary to accumulate enough thioester for reliable measurement by HPLC while also keeping [cPGA] well below K_m throughout the kinetic experiment. In this hypothetical example, the presence of 30 nM of DJ-1 reduced the concentration of thioester from 85 to 35 µM indicating that 50 µM was converted by DJ-1 into 3-PG. The ratio of [3PG] to [thioester] is the same as the ratio of reaction rates, allowing the deduction of k_{cat}/K_m.

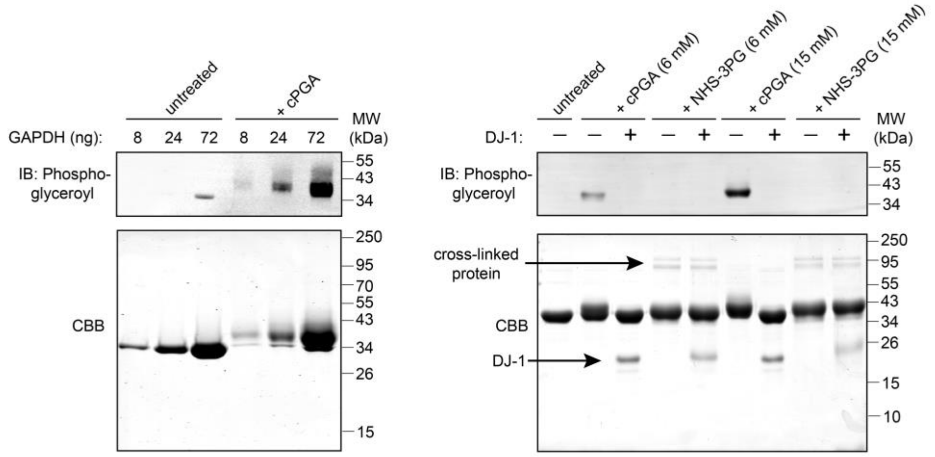

Supplementary Figure 8. Validation of anti-3PG antibodies and evaluation of 3PG conjugation procedures. *(left)* GAPDH at 5 mg/ml was treated with 17 mM cPGA and used for immunization. The same sample was immunoblotted at indicated amounts to test rabbit serum 4 weeks after the first immunization. The results suggest that most antibodies recognize 3PG-modified GAPDH and not unmodified GAPDH. A small fraction of antibodies that recognize unmodified GAPDH was later removed by passing antibody solution through the column with immobilized GAPDH. Coomassie stained gel below shows the same samples but at 100x concentration, i.e. 0.8, 2.4 and 7.2 µg of GAPDH. *(right)* GAPDH (1 mg/ml in 0.1 M sodium phosphate buffer pH 7.6) was modified either by cPGA or according to the published protocol[1] that uses 15 minute pre-incubation of 3PG with a large excess of EDC and sulfo-NHS before protein modification. In both cases a complete conversion of 3PG into either cPGA or NHS ester was presumed. Both protocols result in an upward band shift visible on Coomassie stained gel (4 µg of GAPDH per lane) suggesting modification. Comparison with samples containing DJ-1 (0.1 mg/ml) suggests that modification by the EDC/NHS protocol is not mediated by cPGA. Immunoblot analysis of the same samples (200 ng of GAPDH per lane) with anti-pgK antibodies reveals that cPGA treatment resulted in an expected modification, but the EDC/NHS protocol did not produce any signal. The absence of 3PG signal in samples produced with the regular EDC/NHS protocol is likely because 3PG is quickly converted to 2,3-phosphodiester of glyceric acid by excess EDC which reacts with excess EDC again and only then modifies GAPDH. Thus, it is likely that the two protocols result in modification of GAPDH by different molecules. Blots and gels are representative of at least three independent experiments.

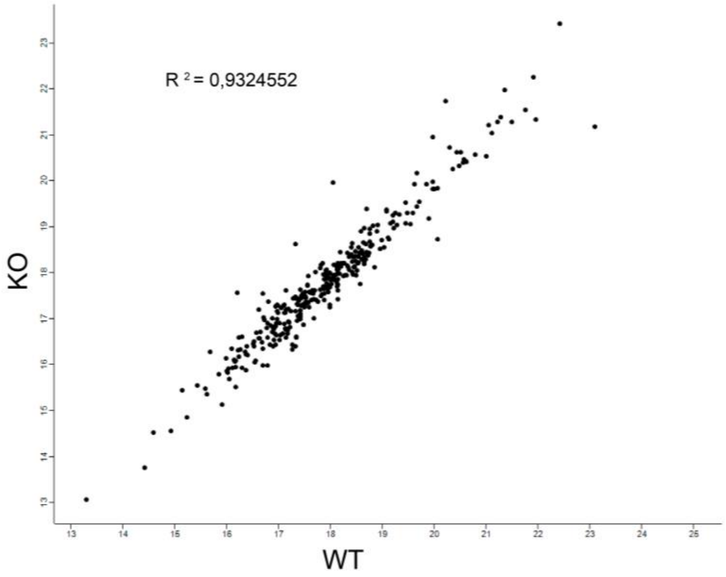

Supplementary Figure 9. Correlation between the label-free quantification values. $\log_2$ LFQ intensities of each identified protein in WT and KO samples are shown. This plot is representative of three independent experiments.

Uncropped images for Supplementary Figure 8

Top left:

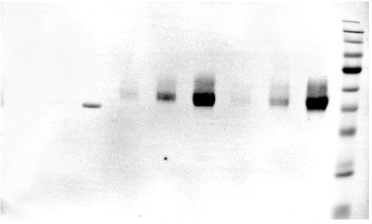

Bottom left:

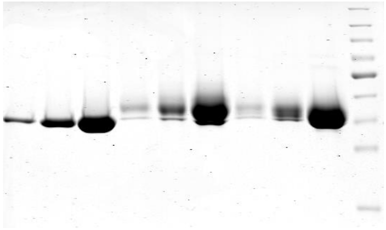

Top right:

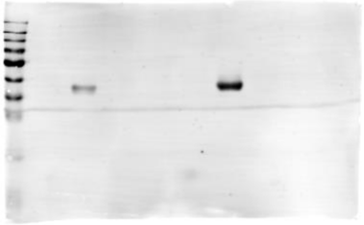

Bottom right:

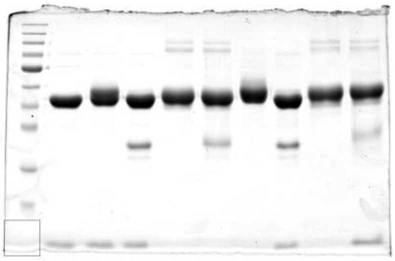

Supplementary References

1. Moellering, R.E. & Cravatt, B.F. Functional lysine modification by an intrinsically reactive primary glycolytic metabolite. *Science* **341**, 549-53 (2013).
