# Supplementary material for: DJ-1 protects proteins from acylation by catalyzing the hydrolysis of highly reactive cyclic 3-phosphoglyceric anhydride
Source: Nat Commun. 2024 Mar 5;15:2004. doi: 10.1038/s41467-024-46391-9 (PMC10915168; doi:10.1038/s41467-024-46391-9)
Supplement: Supplementary file 3 — Description of Additional Supplementary Files [file 41467_2024_46391_MOESM3_ESM.pdf]

File Name: Supplementary Data 1

Description: Proteomics data
