# Supplementary material for: DJ-1 protects proteins from acylation by catalyzing the hydrolysis of highly reactive cyclic 3-phosphoglyceric anhydride
Source: Nat Commun. 2024 Mar 5;15:2004. doi: 10.1038/s41467-024-46391-9 (PMC10915168; doi:10.1038/s41467-024-46391-9)
Supplement: Supplementary file 5 — Reporting Summary [file 41467_2024_46391_MOESM5_ESM.pdf]

nature portfolio

Corresponding author(s): Darkhan Utepbergenov

Last updated by author(s): Feb 5, 2024

Reporting Summary

Nature Portfolio wishes to improve the reproducibility of the work that we publish. This form provides structure for consistency and transparency in reporting. For further information on Nature Portfolio policies, see our Editorial Policies and the Editorial Policy Checklist.

Statistics

For all statistical analyses, confirm that the following items are present in the figure legend, table legend, main text, or Methods section.

n/a	Confirmed	
☐	☒	The exact sample size (*n*) for each experimental group/condition, given as a discrete number and unit of measurement
☐	☒	A statement on whether measurements were taken from distinct samples or whether the same sample was measured repeatedly
☐	☒	The statistical test(s) used AND whether they are one- or two-sided<br>*Only common tests should be described solely by name; describe more complex techniques in the Methods section.*
☒	☐	A description of all covariates tested
☒	☐	A description of any assumptions or corrections, such as tests of normality and adjustment for multiple comparisons
☐	☒	A full description of the statistical parameters including central tendency (e.g. means) or other basic estimates (e.g. regression coefficient) AND variation (e.g. standard deviation) or associated estimates of uncertainty (e.g. confidence intervals)
☐	☒	For null hypothesis testing, the test statistic (e.g. *F*, *t*, *r*) with confidence intervals, effect sizes, degrees of freedom and *P* value noted<br>*Give P values as exact values whenever suitable.*
☒	☐	For Bayesian analysis, information on the choice of priors and Markov chain Monte Carlo settings
☒	☐	For hierarchical and complex designs, identification of the appropriate level for tests and full reporting of outcomes
☒	☐	Estimates of effect sizes (e.g. Cohen's *d*, Pearson's *r*), indicating how they were calculated

Our web collection on statistics for biologists contains articles on many of the points above.

Software and code

Policy information about availability of computer code

Data collection	NMR spectroscopy data was collected using FT NMR Spectrometer Jeol JNM ECA-500 (JEOL), Delta (v5.0.4).<br>MS data were collected using Impact II QTOF tandem mass spectrometer (Bruker Daltonics) using Bruker qtofControl Version v4.0.<br>Mass spectrometric detection was performed using Agilent 6530 accurate-mass Q-TOF LC/MS system with agilent 1290 infinity operating in negative electrospray ionization mode.<br>UltiMateTM 3000 UHPLC (Thermo ScientificTM) controlled by the Chromeleon 7.2 software was used for data acquisition, integration of chromatograms, calibration curve calculation, and<br>quantification of study samples.<br>Optical absorbance data was collected using UV-2600i UV-Vis spectrophotometer (Shimadzu).<br>Image Studio (v2.1.10) was used to collect Western blot data from Odyssey CLx infrared imaging system (LI-COR).
Data analysis	Mass Spectrometry data was analyzed using Bruker Compass Data Analysis (v4.3), MaxQuant (v2.4.0.0), Perseus (v2.0.10.0). Kinetic experiments were analyzed using Kintek global kinetic explorer. Western blotting images were analyzed using Image Studio (v2.1.10).<br>Statistical analysis was performed using the GraphPad Prism 8.

For manuscripts utilizing custom algorithms or software that are central to the research but not yet described in published literature, software must be made available to editors and reviewers. We strongly encourage code deposition in a community repository (e.g. GitHub). See the Nature Portfolio guidelines for submitting code & software for further information.

Data

Policy information about availability of data

All manuscripts must include a data availability statement. This statement should provide the following information, where applicable:

- Accession codes, unique identifiers, or web links for publicly available datasets
- A description of any restrictions on data availability
- For clinical datasets or third party data, please ensure that the statement adheres to our policy

The mass spectrometry proteomics data have been deposited to the ProteomeXchange Consortium (http://proteomecentral.proteomexchange.org) via the iProX partner repository with the dataset identifier PXD043887 [https://proteomecentral.proteomexchange.org/cgi/GetDataset?ID=PXD043887]. Raw NMR spectra are available from the corresponding author upon request. Source data are provided with this paper. Raw NMR spectra are available from the corresponding author upon request. The remaining data are available within the Article, Supplementary Information, Supplementary Data or Source Data file. Source data are provided with this paper.

Research involving human participants, their data, or biological material

Policy information about studies with human participants or human data. See also policy information about sex, gender (identity/presentation), and sexual orientation and race, ethnicity and racism.

Reporting on sex and gender	N/A
Reporting on race, ethnicity, or other socially relevant groupings	N/A
Population characteristics	N/A
Recruitment	N/A
Ethics oversight	N/A

Note that full information on the approval of the study protocol must also be provided in the manuscript.

Field-specific reporting

Please select the one below that is the best fit for your research. If you are not sure, read the appropriate sections before making your selection.

☒ Life sciences ☐ Behavioural & social sciences ☐ Ecological, evolutionary & environmental sciences

For a reference copy of the document with all sections, see nature.com/documents/nr-reporting-summary-flat.pdf

Life sciences study design

All studies must disclose on these points even when the disclosure is negative.

Sample size	No statistical calculations were performed to determine the required sample size. After optimization of conditions through pilot experiments, successful experiments were repeated at least twice to ensure reproducibility. Since all experiments could be accurately reproduced at least twice, sample size equals 3 for nearly all of our experiments.
Data exclusions	No data were excluded from this manuscript.
Replication	All experiments of this study are from a minimum of three independent experiments, unless specifically stated. All attempts at replication were successful.
Randomization	Since randomization is used mainly to remove selection and/or accidental bias, it is not required (or not applicable) for most of our experiments that use pure chemicals and purified proteins (i.e. have strictly defined chemical composition).<br>A couple of experiments that do involve biological material (e.g. cells) have a potential to be influenced by confounding factors such as covariance. We dealt with these potential problems according to common standards in cell biology research. For example, we selected two independent DJ-1 knockout clones (these clones were produced with different sgRNA sequences to minimize off-target effects) and showed that both clones display a strong difference from wild type cells (e.g. Fig. 4g) while behaving similarly to each other. We also performed additional experiments to test our hypothesis, for example adding protein purified from bacteria to knockout cell cytoplasm (Fig. 4g).
Blinding	A blind was not imposed on researchers as blinding is uncommon for biochemical and chemical experiments.

Reporting for specific materials, systems and methods

We require information from authors about some types of materials, experimental systems and methods used in many studies. Here, indicate whether each material, system or method listed is relevant to your study. If you are not sure if a list item applies to your research, read the appropriate section before selecting a response.

Materials & experimental systems

n/a	Involved in the study
☐	☒ Antibodies
☐	☒ Eukaryotic cell lines
☒	☐ Palaeontology and archaeology
☐	☒ Animals and other organisms
☒	☐ Clinical data
☒	☐ Dual use research of concern
☒	☐ Plants

Methods

n/a	Involved in the study
☒	☐ ChIP-seq
☒	☐ Flow cytometry
☒	☐ MRI-based neuroimaging

Antibodies

Antibodies used	Primary antibodies:<br>Anti-acetylated alpha Tubulin Antibody (Santa Cruz Biotechnology, catalog no.sc-23950, lot no. E2722, diluted to 1:1,000)<br>Anti-DJ-1 rabbit polyclonal antibodies (National Centre of Biotechnology, Astana, Kazakhstan, 2mg/mL, diluted to 1:1,000)<br>Anti-pgK rabbit polyclonal antibodies (National Centre of Biotechnology, Astana, Kazakhstan, 2mg/mL, diluted to 1:1,000)<br>Alexa Fluor 680 Anti-GAPDH antibody (Abcam, catalog no. ab184095, lot no.GR3253161-3, diluted to 1:5,000)<br><br>Secondary antibodies:<br>Goat pAb to Rb IgG 680RD (Abcam, catalog no. ab216777, lot no. GR3361088, diluted to 1:20,000)<br>Goat pAb to Ms IgG 800CW (Abcam, catalog no. ab216772, lot no. GR3373742-1, diluted to 1:20,000)
Validation	The commercially available antibodies were validated by each company and validation information can be found at each company's websites.<br>Anti-DJ-1 rabbit polyclonal antibodies were checked and validated in our previous study [Andreeva, A. et al. The apparent deglycase activity of DJ-1 results from the conversion of free<br>547 methylglyoxal present in fast equilibrium with hemithioacetals and hemiaminals. J Biol Chem 294, 548 18863-18872 (2019)].<br>Anti-pgK rabbit polyclonal antibodies were validated and described in the legend of Supplementary Figure 8.

Eukaryotic cell lines

Policy information about cell lines and Sex and Gender in Research

Cell line source(s)	HCT116 cell line used in this study were sourced from ATCC.
Authentication	The cell line was not independently authenticated.
Mycoplasma contamination	The cell line does not have mycoplasma contamination.
Commonly misidentified lines (See ICLAC register)	No commonly misidentified cell lines were used.

Animals and other research organisms

Policy information about studies involving animals; ARRIVE guidelines recommended for reporting animal research, and Sex and Gender in Research

Laboratory animals	Two 4-month old white male New Zealand White rabbits were used for immunization. Animals were maintained in the pathogen-free environment at 21-23C with 60-70% humidity.
Wild animals	The study did not involve wild animals.
Reporting on sex	This is not applicable.
Field-collected samples	The study did not involve field-collected samples.
Ethics oversight	The production of anti-pgK was approved by the Institutional Animal Care and Use Committee (IACUC) of the National center for biotechnology (IRB00013497 National Center of Biotechnology IRB #3). The authors assert that all experiments were performed in

accordance with relevant guidelines and regulations. All research work with laboratory animals was performed in accordance with generally accepted ethical standards and comply with the rules adopted by the European Convention for the Protection of Vertebrate Animals Used for Research and Other Scientific Purposes.

Note that full information on the approval of the study protocol must also be provided in the manuscript.
